# Exploring the benefits and challenges of establishing a DRI-like process for *bioactives*

**DOI:** 10.1007/s00394-014-0666-3

**Published:** 2014-02-25

**Authors:** Joanne R. Lupton, Stephanie A. Atkinson, Namsoo Chang, Cesar G. Fraga, Joseph Levy, Mark Messina, David P. Richardson, Ben van Ommen, Yuexin Yang, James C. Griffiths, John Hathcock

**Affiliations:** 1 Texas A&M University, College Station, TX USA; 2 McMaster University, Hamilton, ON Canada; 3 Ewha Womans University, Seoul, Korea; 4 University of Buenos Aires, Buenos Aires, Argentina; 5 Ben-Gurion University of the Negev and Soroka Medical Center, Beer-Sheva, Israel; 6 Nutrition Matters, Inc., Port Townsend, WA USA; 7 DPR Nutrition Ltd, Surrey, UK; 8 School of Chemistry, Food and Pharmacy, University of Reading, Reading, UK; 9 TNO, Zeist, The Netherlands; 10 China CDC, Beijing, China; 11 Council for Responsible Nutrition, Washington, DC USA

**Keywords:** *Bioactives*, Dietary reference intakes, Non-essential nutrients, Adequate intake

## Abstract

*Bioactives* can be defined as: “Constituents in foods or dietary supplements, other than those needed to meet basic human nutritional needs, which are responsible for changes in health status” (Office of Disease Prevention and Health Promotion, Office of Public Health and Science, Department of Health and Human Services in Fed Reg 69:55821–55822, 2004). Although traditional nutrients, such as vitamins, minerals, protein, essential fatty acids and essential amino acids, have dietary reference intake (DRI) values, there is no such evaluative process for *bioactives*. For certain classes of *bioactives,* substantial scientific evidence exists to validate a relationship between their intake and enhanced health conditions or reduced risk of disease. In addition, the study of *bioactives* and their relationship to disease risk is a growing area of research supported by government, academic institutions, and food and supplement manufacturers. Importantly, consumers are purchasing foods containing *bioactives*, yet there is no evaluative process in place to let the public know how strong the science is behind the benefits or the quantitative amounts needed to achieve these beneficial health effects. This conference, *Bioactives: Qualitative Nutrient Reference Values for Life*-*stage Groups?*, explored why it is important to have a DRI-like process for *bioactives* and challenges for establishing such a process.

## Why it is important to have a DRI-like process for the evaluation of *bioactives*

*Bioactives are important to human health, they are an active area of research, and consumers are purposefully purchasing foods containing them.* Substantial evidence exists that specific *bioactives* beneficially affect health. This conference heard from three experts on *bioactives:* Dr. Cesar G. Fraga on flavanols; Dr. Joseph Levy on lycopene and other tomato carotenoids; and Dr. Mark Messina on soybean isoflavones. A few of their major points are discussed here, since the overall goal of the conference was to discuss the feasibility of applying a dietary reference intake (DRI)-like process to the evaluation of *bioactives* rather than a scientific discussion on *bioactives* themselves, the reader is referred to a number of key papers for more information in support of a specific *bioactive* and decreased risk of a disease or other health-related condition. There is strong evidence for the effect of flavanols on decreased risk of cardiovascular disease and associated risk factors. This evidence includes demographic data and human interventions, and it is mechanistically supported by animal and ex vivo studies [10]. (-)-Epicatechin is the compound better studied [24]; however, other flavanols and flavonoids could share these protective actions [30]. Another important class of *bioactives* is isoflavones derived from soybean. Although it is not possible to infer a direct causal relationship, case–control and prospective epidemiologic studies show isoflavone intake via soyfoods is associated with a lower risk of several chronic diseases including breast [32] and prostate [33] cancer, and among women, coronary heart disease (CHD) [18] and osteoporosis [17, 34]. Furthermore, there is relatively solid evidence that isoflavones increase flow-mediated dilation in post-menopausal women with impaired endothelial function [6] and there is suggestive, but limited evidence, that isoflavones reduce carotid intima media thickness [12]. The most impressive clinical data exist for the alleviation of menopausal hot flashes [27]. Lycopene and other tomato carotenoids have been found to decrease blood pressure in pre-hypertensive patients as well as reduce post-prandial blood-oxidized low-density lipoproteins [5, 7, 16, 22].

Research on *bioactives* is a significant portion of diet, nutrition and disease portfolios of governments, at universities, and at food manufacturers. Consumers are interested in optimal health and are purposefully purchasing foods containing *bioactives*. However, there is no evaluative process in place to inform the public about the strength of the science behind the purported benefits of a specific *bioactive* of interest, nor is there information on how much of a particular *bioactive* is necessary to be of benefit. If there were a process to evaluate the strength of the science behind the intake of a *bioactive* and decreased risk of disease (or other health condition), standards would be set for this research, studies could be compared across laboratories, and consumers and health professionals could have more confidence in what they were eating; and the field could move forward more quickly. If that science base were combined with a recommended intake value, assessments could be made as to whether or not populations or specific age groups were meeting that recommendation and consumers would know the overall contribution of a food product to the recommended amount.

*Having a DRI value increases the status of a bioactive and makes it part of nutrition public policy.* Without a DRI value, it is unlikely that *bioactive* information will be incorporated into national nutrition intake surveys such as NHANES (National Health and Nutrition Examination Survey) in the US. National nutrition surveys describe the amount of nutrients being consumed by representative populations, and then those intake values are compared to a DRI to determine whether the population is eating too much or too little of that substance. If too little, the substance might be called an “at risk” nutrient, and education campaigns to improve people’s intakes (within one’s calorie allotment) could be implemented. Thus, not having reference intake values limits the ability to develop messaging to the public regarding *bioactives* for which there is solid scientific evidence of their health-enhancing effects. Importantly, health professionals (such as physicians, physician assistants, nurses, and dietitians) who may offer advice to clients on what they should be eating, would be more comfortable recommending *bioactives* if they have gone through a rigorous evaluation process. In most countries, the overall nutrition policy is called “dietary guidance”. Although this guidance is food rather than nutrient based, the food recommendations are derived from the DRI values for the nutrients. For example, the philosophy of the US Dietary Guidelines is that if one follows the recommendations of the guidelines, one will automatically meet the DRI values for all nutrients [20]. Thus, dietary guidance is another important way that information on *bioactives* with substantial science behind their efficacy could be transmitted to consumers. For a summary of the advantages of having a DRI-like process for the evaluation of *bioactives*, see Table 1.

**Table 1:** Why it is important to have a DRI-like process for the evaluation of *bioactives*

Importance	Example	Benefit for having a DRI-like value
*Bioactives* are important to human health	For example, there is strong science behind the relationship between flavanols and decreased risk of cardiovascular disease [10, 15]; isoflavones and lower risk of several chronic diseases [18, 27, 32]; and lycopene and other tomato carotenoids and decreased blood pressure [5, 7, 16, 22]	A major benefit would be that they would be recognized as being important to health and evaluated accordingly. Investigators, regulatory agencies, consumers would all know how strong the science was behind science messaging on these compounds
*Bioactives* are a significant portion of diet and disease research portfolios	Governments, Universities, and Food Manufacturers are supporting studies on *bioactives*	Standards would be set so that studies could be compared across laboratories
Consumers are interested in optimal health and are purposefully purchasing foods containing *bioactives*	This was part of the rationale for setting DRI values for *bioactives* in China	Consumers would benefit from strengthened knowledge that they were making decisions based on science and they would also have a target to aim for in terms of intake
Having a DRI value increases the status of a bioactive and makes it part of nutrition public policy	Substances that have DRI values are regularly evaluated in populations to see if that population is meeting established DRI values	If the bioactive is part of the intake assessment of nutrients/foods, then we will learn whether or not that population is actually meeting the DRI value, or if it is an “at risk” nutrient
The process by which a bioactive is evaluated would set standards which would raise the level of science	Such requirements as having a formal definition, and an approved method of analysis would help comparing studies across laboratories	Using common methods of analysis and a common definition would allow studies to build on each other and advance the science more rapidly
With a transparent process for evaluation, the results would provide science-based recommendations for improving diets	Health professionals such as doctors, dietitians, and educators would be more comfortable making diet recommendations	Messaging on intake of *bioactives* would be science based
Having an intake value would set a goal for incorporating *bioactives* into diets	Consumers would know whether a food was a good source of that bioactive, or how much one would need to eat in order to reach the intake value	Having a target intake value would discourage messaging on products that suggest they are a good source of a specific bioactive when they only contain a negligible amount

*Dietary fiber is an example of a bioactive with a DRI value.* Although dietary fiber is a non-essential nutrient, it does have an officially recommended intake value [14]. This means that the amount of fiber in a food product is on most fact-based food labels throughout the world. It is also generally included in the questionnaires on national food intake surveys so that information is available as to whether or not the DRI value for fiber is being met. It also means that it is considered and promoted in dietary guidance. Dietary fiber is thus of concern to consumers who are looking to increase it in their diets.

## What are the challenges to establishing such a system and how can those challenges be met

The process for determining nutrient reference values in the US and Canada changed significantly in 1994 when several kinds of reference values were introduced and articulated in the 1994 publication, *How Should The Recommended Dietary Allowances Be Revised* [9]. There were two major changes: (1) that values could be based on reduced risk of a disease and (2) that there were additional values other than the recommended dietary allowance (RDA), i.e., estimated average requirement, adequate intake (AI), and upper level (UL). A conclusion of this report was that the “reduction in risk of chronic disease is a concept that should be included in the formulation of future RDAs where sufficient data for efficacy and safety exist [9] ”. This conclusion represented a “new paradigm” from what had previously existed. Using these criteria, four DRI values have since been set based on chronic disease: osteoporosis and fractures for calcium and vitamin D, dental caries for fluoride, CHD for fiber, and a combination of endpoints including salt sensitivity, kidney stones, and blood pressure for potassium [28]. Thus, this suggests that *bioactives* could qualify for a DRI value if they could show strong science behind reduced risk of disease.

*Demonstrating reduced risk of disease with a bioactive is more difficult than it is to show prevention of a deficiency outcome with an essential nutrient.* A major difference between *bioactives* and essential nutrients (i.e., vitamins, minerals, essential fatty acids, and essential amino acids) is that the absence of *bioactives* in the diet does not result in a deficiency disease, whereas the absence of an essential nutrient eventually results in deficiency symptoms (e.g., lack of vitamin C and scurvy, thiamin and beriberi, iron and anemia). This difference means that a DRI value would have to be based on an endpoint other than a deficiency disease. As shown above, this could be decreased risk of a chronic disease, but showing cause and effect with a *bioactive* and chronic disease is more difficult than when the disease is specific nutrient related. In other words, if vitamin C intake is inadequate, 100 % of the deficient people will eventually get scurvy. This is not the case for chronic disease which is affected by multiple nutrients, and is also impacted by other non-nutrient factors (e.g., gender, age, and genetics) [28].

Dr. Ben van Ommen challenged the concept of relating health to just decreased risk of disease and suggested that in quantifying the health effects of *bioactives* “we might need to consider in greater depth what health is, what mechanisms are involved in maintaining health, and how to best quantify these”. A pioneer in this new area of “optimal health,” he considers health to be appropriate adaptation to a continuously changing environment—and food is a key part of that changing environment. He calls this adaptive capacity “phenotypic flexibility” and states that it is key to maintenance of overall homeostasis “and thus to a healthy life”. He and his research group have also developed ways to test for “phenotypic flexibility” by stressing specific components of the system that maintain homeostasis and evaluating the stress response reactions. These response reactions usually appear to be more informative and sensitive than their homeostatic counterpart. A classic example is the oral glucose tolerance test versus fasting glucose, and numerous other comparable “challenge biomarkers” that are now being developed [23, 29]. If accepting decreased risk of disease as an endpoint for a DRI value was a paradigm shift, Dr. van Ommen’s emphasis on “phenotypic flexibility” is definitely a new paradigm shift which should become more widely accepted as an evaluation of efficacy for a *bioactive* as the research to measure this flexibility is validated.

### Issues regarding the setting of life-stage DRI values for bioactives

Dr. Stephanie Atkinson discussed possible approaches for determining life-stage DRI values for *bioactives* using information from previous DRI recommendations developed for infants, children, and youth as an example [1]. She suggested using three age groupings to establish DRI values for *bioactives*: (1) infants to 1 year of age; (2) children 1–8 years; and (3) individuals over 8 years. For Infants to 1 year of age, she suggested using human milk as a “reference”. For children 1–8 years, in the absence of clinical trials, it was suggested that AI values be derived from population-based intake data associated with health outcomes. For those over age eight, the suggestion was to derive the value from existing data on biomarkers of chronic disease or extrapolation from adults. The rationale and the cautions for each of these recommendations were provided. Issues in establishing life-stage DRIs for *bioactives* vary greatly from one substance to another. For example, in infants, intakes from human milk *bioactive* substances such as nucleotides [25], carnitine [2], lutein [3], and glycoconjugate sugars [19, 26] have been used to derive safe levels of addition of such compounds to infant formulas. Evidence of the biological benefit of addition of these substances to the health of formula-fed infants is inconsistent, but no adverse effects have been identified. A lack of response to addition of a *bioactive* to formula may relate to the variable bioavailability of a *bioactive* depending on whether it is found in breast milk or added to formula. For example, approximately four times more lutein is needed in infant formula than is naturally present in human milk to achieve similar infant serum lutein concentrations [3]. For the case of dietary fiber, using human milk as a reference for infants 7–12 months of age cannot be done because of the absence of this substance in milk. Remaining challenges include selection of the best model (approach): For example, objectively differentiating between the various age groups on a basis other than age itself seems logical, if difficult. Also, the development of recommended intakes or maximal effect ranges is another choice.

### Establishing safety of *bioactives* and adjusting for different population groups may not be the same as it is for essential nutrients

Dr. David Richardson discussed the process of establishing the safety of *bioactives*. For nutrients and other dietary ingredients, the limitations on safety are commonly set through identifying a “Tolerable Safe UL”. This is done by identifying any “hazard” associated with high intakes, establishing a dose–response relationship, evaluating the uncertainty and selecting a composite “safety factor,” and then calculating an UL value. This procedure cannot be applied when no hazard can be identified (as with many *bioactives*). However, there is an alternative risk assessment approach that is based on the highest observed intake (HOI) method developed by FAO/WHO [8] and included in Codex Guidelines [6]. The HOI is defined as the highest level of intake observed with the available data of acceptable quality, showing an absence of adverse effects. Since most *bioactives* have no known hazard, the HOI is an important alternative approach to setting quantitative value limits on the amounts of *bioactives* that may be considered safe.

Even if there is agreement on this general approach, the problem remains that most safety data are derived from studies on adult subjects designed to look for benefit rather than harm. Scaling the healthy adult values to give confident estimates of the amounts to be deemed safe in subpopulation groups is difficult. Nonetheless, an adult UL or HOI value is needed to give an appropriate basis for policies directed to other population groups. Most DRI values fall well below the ULs/safe ULs, but some high intakes can approach or exceed the safe UL. A narrow range between a DRI and upper safe level may be unjustified when there is a lack of evidence of a demonstrable adverse effect/toxicity at current levels above an upper safe level. If intakes exceed the UL/HOI, the significant uncertainties about the safe level are more likely to indicate that the intake is not the problem but rather the application of a safe level based on inadequate data. In practical terms, adverse effects are more often observed with inadequate intakes rather than excessive intakes. Clearly, care and scientific judgment must be taken in the use of a safe UL as the benchmark in the selection of ULs/HOIs for *bioactives.*

### A sustainable approach is needed for the evaluation of efficacy and intake recommendations for *bioactives*

#### Lessons learned from South Korea

There is growing interest in establishing a DRI-like system for setting intake values for *bioactives* [4, 11]. Although South Korea does not have a DRI system for establishing intake values for *bioactives*, they do have an evaluative process together with a process to determine intake values. Dr. Namsoo Chang explained this process for South Korea. The Health Functional Food (HFF) Act was enacted in 2004 with the goal of ensuring the safety of HFF with certain health claims for consumer information. At its inception the HFF covered products in the form of tablets, capsules, powders, granules, pastes, gels, jellies, and bars that were intended to enhance and preserve human health and contained one or more functional ingredients or constituents. In 2008, the scope was extended to include conventional foods and other diet supplements.

What is unique about the HFF act in South Korea is that unlike other countries, the government of South Korea is endorsing a particular product with a HFF “seal”. There are two types of HFF, generic and product-specific. The generic type (shown in Table 2) contains both 28 essential nutrients and 55 non-nutrients. Both the nutrients and non-nutrients are considered to have substantial efficacy and safety data to have been considered for the generic category. All of these substances listed on the generic health/Functional Food Code include health claims and intake recommendations. This generic type HFF is most analogous to establishing a process for evaluation of efficacy and intake values for DRIs, although they are not called DRIs by the South Korean Government.

**Table 2:** Functional ingredients listed in the South Korean Health/Functional Food Code (Generic Type)

Nutrients	Non-nutrients	
Vitamin A	Alkoxyglycerol	Banaba leaf extract
Vitamin D	Aloe gel	Evening primrose seed extract
Vitamin E	Aloe whole leaf	Ganodermalucidun fruit body extracts
Vitamin K	Chitosan/chitooligosaccharide	Garciniacambogia extract
Beta carotene	Chlorella	Ginko leaf extract
Vitamin B1	CLA	Green tea extracts
Vitamin B2	Coenzyme Q10	Guava leaf extract
Vitamin B6	Fructooligosaccharide	Haematococcus extract
Vitamin B12	Gamma-linoleic acid	Japanese apricot extract
Niacin	Ginseng	Milk thistle extract
Vitamin C	Glucosamine	Propolis extract
Pantothenic acid	L-theanine	Saw palmetto extract
Folic acid	Lecithin	Functional fiber
Biotin	Lutein	Guar gum/hydrolyzates
Calcium	MSM	Glucomannan
Magnesium	Mucopolysaccharide	Indigestible maltodextrin
Potassium	N-acetylglucosamine	Oat fiber
Zinc	Octacosanol	Soy fiber
Copper	Omega-3 fatty acids	Tree ear
Selenium	Phosphatidylserine	Wheat fiber
Manganese	Phytosterol ester	Barley fiber
Iron	Plants containing chlorophyll	Arabic gum
Iodine	Probiotics	Corn bran
Molybdenum	Red ginseng	Inulin
Chrome	Red yeast rice	Psyllium husk
Dietary fiber	Soy isoflavone	Polydextrose
Essential fatty acids	Soy protein	Fenugreek seed
Protein	Spirulina	
	Squalene	

If the *bioactive* is not on the generic type list of functional ingredients, then it needs to follow a process and receive approval. Manufacturers submit a dossier for comprehensive scientific evaluation of safety and efficacy, which is reviewed by the Government and Advisory Committees. The application must consist of any data on the history of safe use, manufacturing processes, recommend intake levels, toxicological data, clinical data, nutritional evaluation data, and bioavailability data.

Soy isoflavones (discussed in this conference) have a generic health claim which is that they help to maintain bone health. The isoflavone content of common soybean products in Korea is known, as is the isoflavone intake in South Korea. Although no safety data were available in Korea, the safe intake level for isoflavones was adopted from the Japanese standards. A recommended intake is set at 24–27 mg/day as aglycone soybean isoflavones. Notably, a caution is stated for infants, children, pregnant and lactating women, and individuals who have an allergy to soybean, and individuals who are sensitive to estrogen. A generic claim for lutein (a *bioactive* found in tomatoes) also exists. The health claim is, “helps eye health by maintaining the density of macular pigments which can be decreased by aging”. Based on review of existing literature, the intake recommendation was set at 10–20 mg lutein/day with a warning for yellowing of skin if taken at excessive amounts. In addition, a recommendation for intake of all-*trans* lycopene at 5.7–15 mg/day is provided based on the health claim for tomato extracts as an antioxidant. This recommendation is accompanied by a caution for pregnant and lactating women and for children. Flavonoids and lycopene are listed in the product-specific category, rather than the generic category. A flavonoid database is available for commonly consumed food by Koreans based on the USDA and Japanese flavonoid databases, which were developed in 2009. Flavonoids have been linked to reduced risk for chronic diseases and improved health outcomes, and six subclasses of flavonoids are identified by structure.

Currently, the Ministry of Health and Welfare is revising the South Korean DRIs and plans to release the revised version in 2015. Although it was recently decided that *bioactive* substances will not be included in the 2015 version of the DRIs, the need to establish DRIs for *bioactive* substances was raised. If there were to be a DRI value for *bioactives*, it would most likely be the AI value. The AI is defined as “The recommended average daily intake level based on observed or experimentally determined approximations or estimates of nutrient intake by a group (or groups) of apparently healthy people that are assumed to be adequate—used when an RDA cannot be determined” [13]. Importantly, South Korea may be able to contribute to establishing ULs for *bioactives,* since they have a post-market surveillance system on health/functional foods. They are operating an online system for adverse events data collection from consumers, manufacturers, and healthcare professionals. They have the integrated database on products and safety data and are in the process of doing statistical modeling to determine a cause effect relationship of any adverse event.

#### Setting specific proposed levels for bioactive compounds: Recent experiences in China

Professor Yang Yuexin described the process for setting a special category of DRIs (called specific proposed level; SPL) in China. This new category is used to evaluate and assign an intake value for *bioactives*. This is the only country, of which we are aware, that has actually established DRI values for *bioactives.* The China Nutrition Society, similar to the Institute of Medicine in the US, changed their intake evaluation process for nutrients from only RDAs to DRIs. This change was initiated in 2000 and resulted in 32 DRI values for nutrients. In 2010, they initiated the incorporation of a SPL for non-nutrients and a proposed Intake that is based on reducing the risk of non-communicable chronic disease and improving optimal health. Their stated rationale as to why they consider the SPL a DRI value is that both traditional medicine and modern nutrition research have deepened the understanding of plant compounds; and also because consumers are widely consuming these bioactive substances in China. In 2010, they had seven different expert review panels containing a total of 87 experts develope the DRIs for China to be released in 2014. One of the seven panels was on “non-nutrients,” and 21 experts were involved in this panel. The goal of this panel was to develop DRI values for water, fiber, and 18 phytochemicals (SPLs). The SPLs reflect the current state of scientific knowledge and are published as a series of reports by the Chinese Nutrition Society. Both SPLs and ULs are set for *bioactives*. Table 3 shows the “non-nutrients” that were evaluated by the Chinese DRI process.

**Table 3:** “Non-nutrients” that were evaluated by the Chinese DRI Process

Non-nutrient substances	
Water	Lycopene
Dietary fiber	Proanthocyanidines
FOS anthocyanin	Isoflavones
Resveratrol	Phytosterols
Catechol	Isothiocyanates
Quercetin	Allicin, gallic
Curcumin	Glucosamine
Chlorogenic acid	GABA
Lutein/zeaxanthin	Alpha lipoic acid (LA)
	*L*-carnitine

The Chinese Nutrition Society has acknowledged that there are some *bioactives* that “like some other nutrients, are essential for reaching the full (genetically-determined) lifespan”. They have termed these nutrients as “life span essential” [31]. The Chinese experience in establishing DRI-like values for *bioactives* should be followed closely, and they should be acknowledged as being the pioneers in this area.

### Setting a high bar for entrance into the evaluation system

One issue with setting up a DRI-like process for the evaluation of *bioactives* is the very wide range of the strength of the science behind the intake of a *bioactive* and a purported reduced risk of disease. For some *bioactives*, little research has been conducted, whereas for others there are 20–30 years of research in support of a protective effect. A concern is that the evaluators would have to be dealing with requests when there was insufficient information to apply the process. One suggestion to offset this challenge is to set a high standard for “entrance into the evaluative process”. Dr. Joanne Lupton discussed potential entrance criteria as necessary information before a *bioactive* could be considered for a DRI-like evaluation process (see Table 4). Setting these nine criteria as essential for consideration for evaluation serves several goals: It minimizes the effort of the evaluator; and importantly, it sets a standard, if met, that investigators and funding sources could design their research to meet, knowing that there would be a certain level of credibility if they were to do so.

**Table 4:** Proposed criteria for a bioactive to qualify for evaluation

Criterion	Additional information	Rationale for criterion
A definition of the substance which is commonly accepted	Definition should match the method of analysis	Makes it easier to build a database of efficacy of bioactive if substances with the same definition are compared
A method of analyzing the substance which is consistent with the definition	Preferably backed up by a multi-center analysis such as an AOAC method	Facilitates comparing studies across laboratories. Need a definition and an approved method of measuring so that intake values can be determined, and if populations are meeting recommended intake values
Database of the amount of the bioactive in foods	Preferably global and updated on a regular basis as new foods come on the market	To determine the amount of this bioactive currently in the food supply and enable determining how much people are consuming. Also necessary for baseline data for clinical trials and input into epidemiological studies
Prospective cohort studies	Both sexes, showing decreased risk of a disease such as CVD with increased intake of the bioactive. Must be able to isolate the specific bioactive versus other *bioactives*. Best if the bioactive is also measured in blood/urine, etc. in subset of population and supports food intake data. Relationship to the disease should be consistent with clinical trials	Dose–response data or at least highest quintile versus lowest quintile for the bioactive will help to set level of efficacy
Clinical trials on digestion, absorption, activation, transport, excretion of the substance	Important to understand the level of absorption and what substances interfere with that absorption, also what the active molecule is and how long it stays in the blood	This information is useful for determining intake and factors that affect intake, transport, activation, etc
Clinical trials on efficacy and dose–response data	Conducted in healthy populations. Bioactive must be measured. Accepted endpoint linked to decreased risk of the particular disease. If surrogate marker, must be “accepted” by regulatory agencies	Need dose–response data to determine the efficacious level, and determine intake values
Safety data at the level of intake that might be anticipated	Ideally would include safety data for special populations such as children, pregnant or lactating women	Need this information even if the bioactive is considered generally regarded as safe (GRAS). GRAS means “safe for intended use”
Systematic Reviews and/or meta analyses showing efficacy	In the US, the Institute of Medicine now requires systematic reviews for setting DRI values (most recent was calcium and vitamin D). The US Dietary Guidelines now requires these also	Having a systematic review that shows efficacy is a real plus and may be necessary, e.g., a Cochrane review. These reinforce the need to have major prospective epidemiological studies and randomized clinical trials
A plausible biological explanation for efficacy	This is not required but is a very large plus if it is available	Scientists/evaluators of the research are more comfortable if there is an explanation, particularly if that explanation is accepted by the scientific community

### Summary, conclusion, and next steps

The speakers were in consensus that providing a framework for the evaluation of *bioactives* could be of benefit to scientists working in this field, to funders of the research, to governments, and importantly to consumers. However, they were also aware of the potential challenges to establishing such a framework. Clearly, there is a difference between determining intake values for essential nutrients and *bioactives*, and thus the basis of the intake value cannot be on a single-nutrient deficiency disease. Nonetheless, other endpoints such as reduced risk of disease may be applicable. Basing a DRI value on reduced risk of disease has been used for four nutrients that have DRI values. Alternatively, the AI value was considered by some to be an appropriate value for consideration as by definition it can reflect the current intake of specific healthy populations. Setting life-stage values for *bioactives* is also a challenge, but Dr. Atkinson suggested a different model for consideration. Instead of concentrating on the *bioactive*, per se, she suggested establishing goals for life stages. For example, for early life, it might be “optimal development” and markers for that could be body composition, or cognitive/behavioral outcomes. For child/adolescent, the DRI value could be based on early biomarkers that are sensitive indicators of chronic disease risk. Then, *bioactives* that were shown to affect those outcomes could receive intake values for that life stage. This model warrants development and consideration. Another challenge is establishing an UL value for *bioactives* in the absence of any evidence of toxicological effects. Here, it appears that there is an extensive literature on risk/benefit systems which should be considered for application to *bioactives.* Finally, the logistics of how to set the framework, who is the “keeper” of the system, and what it would take for a bioactive to be considered in this framework requires serious consideration. A proposed next step would be a workshop with representation from all key stakeholders to discuss the challenges to having a framework for the evaluation of bioactives and how those challenges may be overcome.
